# Re-evaluating the impact of alternative RNA splicing on proteomic diversity

**DOI:** 10.3389/fgene.2023.1089053

**Published:** 2023-02-09

**Authors:** Jeru Manoj Manuel, Noé Guilloy, Inès Khatir, Xavier Roucou, Benoit Laurent

**Affiliations:** ^1^ Research Center on Aging, Centre Intégré Universitaire de Santé et Services Sociaux de l’Estrie-Centre Hospitalier Universitaire de Sherbrooke, Sherbrooke, QC, Canada; ^2^ Department of Biochemistry and Functional Genomics, Faculty of Medicine and Health Sciences, Université de Sherbrooke, Sherbrooke, QC, Canada; ^3^ Centre de Recherche du Centre Hospitalier Universitaire de Sherbrooke (CRCHUS), Sherbrooke, QC, Canada; ^4^ Quebec Network for Research on Protein Function Structure and Engineering, PROTEO, Québec, QC, Canada

**Keywords:** alternative splicing, RNA, isoform proteins, alternative proteins, ghost proteome

## Abstract

Alternative splicing (AS) constitutes a mechanism by which protein-coding genes and long non-coding RNA (lncRNA) genes produce more than a single mature transcript. From plants to humans, AS is a powerful process that increases transcriptome complexity. Importantly, splice variants produced from AS can potentially encode for distinct protein isoforms which can lose or gain specific domains and, hence, differ in their functional properties. Advances in proteomics have shown that the proteome is indeed diverse due to the presence of numerous protein isoforms. For the past decades, with the help of advanced high-throughput technologies, numerous alternatively spliced transcripts have been identified. However, the low detection rate of protein isoforms in proteomic studies raised debatable questions on whether AS contributes to proteomic diversity and on how many AS events are really functional. We propose here to assess and discuss the impact of AS on proteomic complexity in the light of the technological progress, updated genome annotation, and current scientific knowledge.

## Introduction

Alternative splicing (AS) is a key process by which genes produce more than a single mRNA, hence contributing to the transcriptome complexity. In this process, specific exons of a gene can be included or excluded in the final RNA. Protein-coding genes and lncRNA genes can generate multiple splice variants from one gene through AS (Mercer et al., 2011; Khan, Wellinger, and Laurent 2021). From plants to humans, AS is a powerful mechanism that increases transcriptome plasticity and can control the expression level of certain genes (Castle et al., 2008; Gueroussov et al., 2015; Muhammad et al., 2022). Indeed, RNA splice variants arising from AS can exhibit different mRNA stabilities and structures. In humans, it is estimated that 95% of genes undergo AS, which underscores its importance (Castle et al., 2008; Pan et al., 2008; Nilsen and Graveley 2010). Three transcripts are produced in average from each protein-coding gene (Khan, Wellinger, and Laurent 2021). Importantly, splice variants produced from protein-coding genes can potentially encode for distinct protein isoforms. For a given gene, the most expressed transcript is usually defined as coding for the canonical protein. This canonical status is determined based on the transcript expression across different tissues of an organism, the conservation of its exon combination with other species, and/or the existence of a functional role for the protein (Osmanli et al., 2022). Compared to their canonical proteins, isoform proteins can lose or gain certain domains and, therefore, can differ in their functional properties by the alteration of localization signals, sequences for post-translational modifications, or interaction with other proteins (Kriventseva et al., 2003; Stamm et al., 2005; Leoni et al., 2011; Light and Elofsson 2013). Advances in proteomics have shown that the proteome is indeed diverse due to the presence of numerous protein isoforms. For the past decades, with the help of advanced high-throughput technologies such as RNA sequencing (RNA-seq), a full catalog of alternatively spliced transcripts has been established, but the functional significance of most AS events remains still largely unknown. Hence, the identification of numerous alternatively spliced transcripts raises important and debatable questions: how many AS events are real and not mere artefacts of splicing machinery? How many AS products are functional? Does AS really expand proteomic diversity? We propose here to re-evaluate and discuss the impact of AS on proteomic diversity in the light of the technological progress, updated genome annotation, and current scientific knowledge.

## Alternative splicing and proteomic diversity: Two different visions

Whether AS is a major source of proteome complexity has always been a contentious issue in the field. For example, on this debatable question, Benjamin J. Blencowe and Michael L. Tress et al. have mutually expressed their contrasting opinions few years ago (Tress et al., 2017a; Blencowe 2017; Tress et al., 2017b).

Michael L. Tress and colleagues claimed that AS might not be the key to proteome complexity. They argued that most genes only expressed one main transcript across multiple cell lines (Gonzalez-Porta et al., 2013), and hence, one single main protein isoform can be detected by high-resolution mass spectroscopy (Abascal et al., 2015; Ezkurdia et al., 2015). The abundance of alternatively spliced variants identified from more than 100 different tissues at various developmental stages was, therefore, in contrast with the low number of multiple protein isoforms per gene. They found that only 2% of genes had multiple isoform proteins (246 genes with splice event-specific peptide evidence over 12,716 human genes for which at least two peptides have been detected) (Abascal et al., 2015). As few genes provided reliable evidence for more than one isoform, the authors stated that alternative variants were not abundant at the protein level (Tress, Abascal, and Valencia 2017a). One possible reason could be the misidentification of a good peptide spectrum with multiple assigned peaks. However, the discrepancies between transcriptomics and proteomics experiments are difficult to explain solely on a technical issue. They described that alternatively spliced exons were not under selective pressure and are evolving neutrally (Tress, Abascal, and Valencia 2017a). This observation suggested in their opinion that AS events were not evolutionary innovations and that most alternatively spliced variants were not functionally important if translated.

In response to Tress et al. (2017a) and Tress et al. (2017b), Benjamin J Blencowe agreed that AS events were mostly specific to species and, hence, are under relaxed selection pressure (Blencowe 2017). However, he pointed out that even though alternatively spliced transcripts were expressed at lower levels than their corresponding main protein isoforms, it did not mean that these splice variants were not translated or did not have a relevant function in a given cell or tissue type. Blencowe argued that protein abundance was predominantly related to transcript abundance (Liu, Beyer, and Aebersold 2016) and that many splice variants identified by transcriptomics have been detected in polysome fractions and were likely translated (Weatheritt, Sterne-Weiler, and Blencowe 2016). Finally, Blencowe stated that the low detection rate of protein isoforms by LC-MS/MS cannot be interpreted since their identification is limited by the coverage and sensitivity of the technology. Indeed, the peptide number largely exceeds the number of sequencing cycles provided using a mass spectrometer, thereby limiting the detection of splice variants compared to a constitutively expressed sequence (Blencowe 2017).

## Different perspectives: Right and wrong at the same time?

These two visions highlight the AS potential role in proteomic diversity on two different ends of the spectrum. The limitations to the available technology and the scientific knowledge at the time the studies were conducted have potentially skewed the interpretation to opposite ends. In this section, we discuss critical points that should be considered to assess the impact of AS on proteomic diversity.

### Alternative splicing: Real or artefact of splicing machinery?

The widespread presence of alternatively spliced transcripts has raised the question of whether they are artefacts of splicing machinery or have a biological purpose (Graveley 2001). With the high complexity of eukaryotic genes and the level of splice-site conservation, numerous AS events are expected to happen along the processing of pre-mRNAs, regardless of their functional relevance (Modrek and Lee 2002). However, having a reduced fidelity of the spliceosome to promote proteome diversification could be problematic for a cell since basic molecular mechanisms cannot afford to jeopardize levels of essential proteins (Hsu and Hertel 2009). Consequently, high degrees of specificity and fidelity are required for pre-mRNA splicing to ensure the correct expression of critical functional mRNAs. Indeed, even though the frequency of aberrant spliced transcripts varies widely among loci, tissues, and species, the minimum splicing error rate in vertebrates is around 0.1% aberrant transcripts per intron (Skandalis 2016). The spliceosome is extremely accurate in selecting splice junctions with error frequencies as low as one per 10^5^ splicing events (Fox-Walsh and Hertel 2009). This estimation was only performed on specific transcripts (i.e., UBA52, RPL23, HPRT, POLB, and TRPV1), so the extent to which the spliceosome is error-prone remains to be globally assessed. Although the spliceosome is prone to errors, mis-spliced mRNAs can be degraded from cells through nonsense-mediated RNA decay (NMD) or other RNA quality control steps (Saudemont et al., 2017; Garcia-Moreno and Romao 2020). Therefore, the spliceosome is unlikely responsible for generating artefactual splice variants.

### An evolutionary perspective of alternative splicing

The importance and functionality of AS events are often associated on whether these events are conserved during evolution. Generally, 95% of human multiexon genes undergo AS (Pan et al., 2008), but this ratio is 60.7% in the fruit fly (*Drosophila melanogaster*) (Graveley et al., 2011), 25% in the nematode (*Caenorhabditis elegans*) (Ramani et al., 2011), and only 2.9% in the green alga (*Volvox carteri*) (Kianianmomeni et al., 2014). Organisms with more complexity tend to have a higher ratio of AS events. There is a strong positive correlation between the number of unique cell types—referred as organism complexity—and the number of AS events (Chen et al., 2014). The study of the evolutionary landscape of AS over ∼350 million years of evolution in vertebrates showed significant differences in AS complexity among vertebrate species, with primates harboring the highest complexity (Barbosa-Morais et al., 2012; Merkin et al., 2012). These studies demonstrated that the variation in gene expression was conserved at the tissue-specific level, while AS was conserved at the species-specific level, suggesting that AS diverged faster than gene expression. Moreover, AS event types varied in their frequency among different organisms. In animals, exon skipping is the most common AS event, which represents around 50% of all AS events (Pan et al., 2008), while in plants, intron retention is the most abundant AS event type (Reddy et al., 2013). Most AS events have variable tissue specificities and appear to be evolving neutrally (Wang et al., 2008). However, a subset of AS events is conserved between species and displays tissue specificity. For example, around 20% of alternative exons are conserved between humans and mice (Modrek and Lee 2002; Abascal et al., 2015). These conserved events are significantly enriched in genes that function in common biological processes and pathways. Alternative exons in these splicing “networks” allow the tissue-specific rewiring of protein–protein interaction networks (Buljan et al., 2012; Ellis et al., 2012; Irimia et al., 2014; Tapial et al., 2017). Investigating these networks in different tissues and organs has revealed that these conserved isoforms play a prominent role in the regulation of neuronal development (Boutz et al., 2007; Jiao et al., 2008; Laurent et al., 2015; Fiszbein et al., 2016), immunity (Zikherman and Weiss 2008), and muscle differentiation (Nakka et al., 2018). However, this evolutionary conservation does not mean that alternative exons, which are not evolutionarily conserved, are not significant and do not participate in proteomic diversity. These isoforms could be expressed in a lineage-specific manner, or they might have just recently evolved. For instance, the exonization of intronic sequences such as repetitive elements is now widely documented in many genomes. In primate and human genomes, Alu elements are the most abundant transposable elements that can generate new exons (i.e., Alu exons) and lead to novel spliced transcripts (Krull, Brosius, and Schmitz 2005). Ribosome profiling and proteomics data from human tissues and cell lines showed that some Alu-derived exons can be translated and present in human proteins (Lin et al., 2016), suggesting that some Alu exons can contribute to proteomic diversity. However, in primates and humans, the high number and complexity of AS events might not reflect the functional expansion of the transcriptome but could be explained by the nearly neutral theory (Ohta 1992). Weak selection results in an excess of neutral or slightly deleterious mutations, including those affecting AS regulation. A reduction of intron splicing accuracy, mutations introducing cryptic splicing signals, and transposable element insertion events can generate novel AS events that produce non-functional spliced transcripts (Pickrell et al., 2010). Since these mutations are not removed by purging selection, they can persist and some of them can selectively give novel functional entities, for example, AS events that become functional.

### Correlation between transcription and translation

One common argument supporting AS contribution to proteomic complexity is that protein abundance is predominantly related to transcript abundance (Liu, Beyer, and Aebersold 2016). Therefore, even low levels of alternatively spliced transcripts have a chance to be translated into functional proteins. However, there are many regulatory mechanisms that can balance the level of protein expression: the translation rate, the degradation rate, the protein synthesis rate, and transport (Vogel and Marcotte 2012; McManus, Cheng, and Vogel 2015). Different subsets of genes exhibit different types of regulation. At a steady state, mRNA levels correlate with protein levels even during dynamic processes such as proliferation or differentiation (Hsieh et al., 2012; Vogel and Marcotte 2012; Kristensen, Gsponer, and Foster 2013; Li et al., 2014). However, the mRNA levels of some genes are proxies for the corresponding protein levels because of post-transcriptional and translational mechanisms (Liu and Aebersold 2016; Liu, Beyer, and Aebersold 2016). For short-term adaptation such as stress response, the regulation of the transcript level of specific genes is unadapted to the cellular response and post-transcriptional mechanisms (e.g., increase of translation or increase of protein degradation) are thereby more efficient. For instance, changes in the translation rate could positively or negatively affect the mRNA–protein ratio (Lackner et al., 2012; Cheng et al., 2016) and, hence, foster a significant contribution of alternatively spliced transcripts to proteomic diversity.

Another argument supporting AS contribution to proteomic complexity is that many splice variants identified by transcriptomics have been detected in polysome fractions and, hence, are likely to be translated (Weatheritt, Sterne-Weiler, and Blencowe 2016). However, there may be significant levels of alternatively spliced transcripts that do not pass co-translational quality control mechanisms and are degraded. Aberrant polypeptides and mRNAs can be detected and eliminated by mRNA quality control systems while engaging the ribosome (Inada 2017). Because the ribosome has a central role in quality control processes, alternatively spliced transcripts associated with the ribosome are not necessarily translated into proteins.

## What is new on proteomic diversity?

Re-evaluating the impact of AS on proteomic diversity necessitates examining the newest developments in this field of investigation, more specifically the technological progress, the update of genome annotation, and the latest advances in scientific knowledge.

### Technological and technical advances

As highlighted by Blencowe, LC-MS/MS has some limitations in identifying all potential protein isoforms in a complex sample. The number of peptides exceeds the number of sequencing cycles provided using a mass spectrometer, and hence, the detection of alternative splice isoforms present in low quantities is limited and could potentially explain why so few alternative isoforms can be detected in proteomics experiments (Blencowe 2017). To address this issue, the integration of RNA-seq with a data-independent acquisition method acquiring all theoretical spectra has been implemented to reduce peptide mapping uncertainty, improve quantitative accuracy, and detect novel peptides (Liu et al., 2017; Jeong, Kim, and Paik 2018; Agosto et al., 2019). This proteogenomic approach yielded high reproducibility between technical and biological replicates and enabled the quantification of a large fraction of the proteome with quantitative accuracy (Poulos et al., 2020). Another limitation to the detection of alternative splice isoforms is also attributed to enzymes used to digest protein samples. The standard protease used in shotgun proteomics is the trypsin that digests at K or R residues, hereby producing short peptides (around six amino acids) and limiting the proteome coverage and detection of isoform proteins (Wang et al., 2018). Other proteases (e.g., chymotrypsin, LysC, LysN, AspN, GluC, and ArgC) have been used to cover complementary fractions of the proteome and improve the detection of specific peptides (Giansanti et al., 2016). A combination of several enzymes could be the best approach to reach comprehensive peptide identification.

Another challenge is to improve the identification of potentially functional transcripts. The development of long-read sequencing technologies has transformed the field since we can now obtain the entire RNA sequence in a single read (Marx 2023). The full-length transcript recovery and quantification helped advance transcript-level analyses of AS processes, distinguish novel isoform changes, and improve the ability to identify functional isoforms (Uapinyoying et al., 2020; De Paoli-Iseppi, Gleeson, and Clark 2021; Hu et al., 2021; Troskie et al., 2021; Wright et al., 2022). For instance, alternative isoforms and tumor-specific isoforms arising from aberrant splicing during liver tumorigenesis were recently identified by single-molecule real-time long-read RNA sequencing (Chen et al., 2019). Another study combined long-read sequencing with polysome profiling and ribosome foot printing data to predict isoform-specific translational status in the rat hippocampus (Wang X et al., 2019). Indeed, single-molecule sequencing also provides the opportunity to improve ribosome profiling quantification by adapting existing methods for translation studies. For example, quantification of the translation of individual transcript isoforms using ribosome-protected mRNA fragments revealed evolutionary conserved impacts of differential splicing on the proteome (Reixachs-Sole et al., 2020). Finally, the single-cell revolution could also help address more accurately the impact of AS on proteomic diversity. Single-cell differential splicing analyses revealed novel differentially expressed splicing junctions (Liu et al., 2021). Single-cell proteomics is now taking the center stage. Novel quantitative single-cell proteomics approaches are capable of consistently quantifying thousands of proteins per cell across thousands of individual cells using limited instrument time and display ultra-high sensitivity to detect changes in a single-cell proteome (Schoof et al., 2021; Brunner et al., 2022). The technology could be applied for detecting specific protein isoforms in a particular cell type and, hence, could give unprecedented insights into the isoform proteome in health and disease. Interestingly, there are now integrated strategies that can profile single-cell proteome and transcriptome in a single reaction, highlighting the promising potential of highly multiplexed single-cell analyses (Genshaft et al., 2016; Specht et al., 2021).

Finally, an additional challenge is that most proteomic data were focused on the identification of proteins derived from alternatively spliced transcripts in steady-state conditions (Blakeley et al., 2010; Ezkurdia et al., 2012; Alfaro et al., 2017). However, most RNA splicing changes have been associated with changes in physiological conditions (e.g., stress response and hypoxia) or between normal and disease states (Ly et al., 2014). Some studies have also addressed the issue of whether targeted perturbations in RNA splicing patterns manifest as changes in the proteomic composition. For example, by depleting a spliceosome component (i.e., PRPF8) and using quantitative proteomics, it was established that significant changes in RNA relative abundance showed consistent changes in protein production (Liu et al., 2017). Using a similar approach, it would be interesting to determine more broadly how changes in AS for a subset of transcripts reflect in differential protein expression and assess the contribution of AS to proteomic complexity.

### Genome annotation

Historically, mRNAs were defined as monocistronic and expected to encode a single protein. In addition, open reading frames (ORFs) shorter than 100 codons were automatically discarded from genome annotations as proteins of this length were deemed too short to be functional (Cheng et al., 2011). However, the annotation rules have considerably limited the exploration of the proteome. Based on the potential polycistronic nature of genes, a deeper ORF annotation from an exhaustive transcriptome has predicted all possible alternative ORFs (altORFs), which are defined as potential protein-coding ORFs located either in UTRs of transcripts, in alternative reading frames within the coding sequence of mRNAs, or in non-coding RNAs (Samandi et al., 2017; Brunet et al., 2018; Brunet et al., 2019). Numerous altORFs were identified to be both in-frame and out-of-frame of annotated ORFs. Many annotated altORFs are conserved in eukaryotes, suggesting that alternative proteins encoded from these alternative start codons might have a function across species. The community used ribosome profiling to capture all translation events across the genome and confirmed the translation of many altORFs (Bazzini et al., 2014; Ji et al., 2015; Samandi et al., 2017; Weaver et al., 2019). Combined with large-scale proteomics, these studies have led to the identification and functional relevance of alternative proteins translated from many altORFs located within mature transcripts (Saghatelian and Couso 2015; Na et al., 2018; Rothnagel and Menschaert 2018; Orr et al., 2020). Many functional studies showed that alternative proteins play central functions in the maintenance of cellular homeostasis (Delcourt et al., 2018; Cardon et al., 2020; Vergara et al., 2020; Brunet et al., 2021a; Cao et al., 2021; Ichihara, Nakayama, and Matsumoto 2022). In humans, mutations creating or deleting altORFs have been associated with physiopathological conditions such as amyotrophic lateral sclerosis (ALS) (Brunet et al., 2021b), craniofrontonasal syndrome (Tavares et al., 2019), and thrombocythemia (Wiestner et al., 1998). Interestingly, mutations found in cancers that are silent for reference proteins can impact the expression of alternative proteins resulting from the mutated mRNA, suggesting that alternative proteins could be new biomarkers of pathologies (Child, Miller, and Geballe 1999; Liu et al., 1999; Barbosa, Peixeiro, and Romao 2013; Sendoel et al., 2017; Schulz et al., 2018).

**FIGURE 1:**
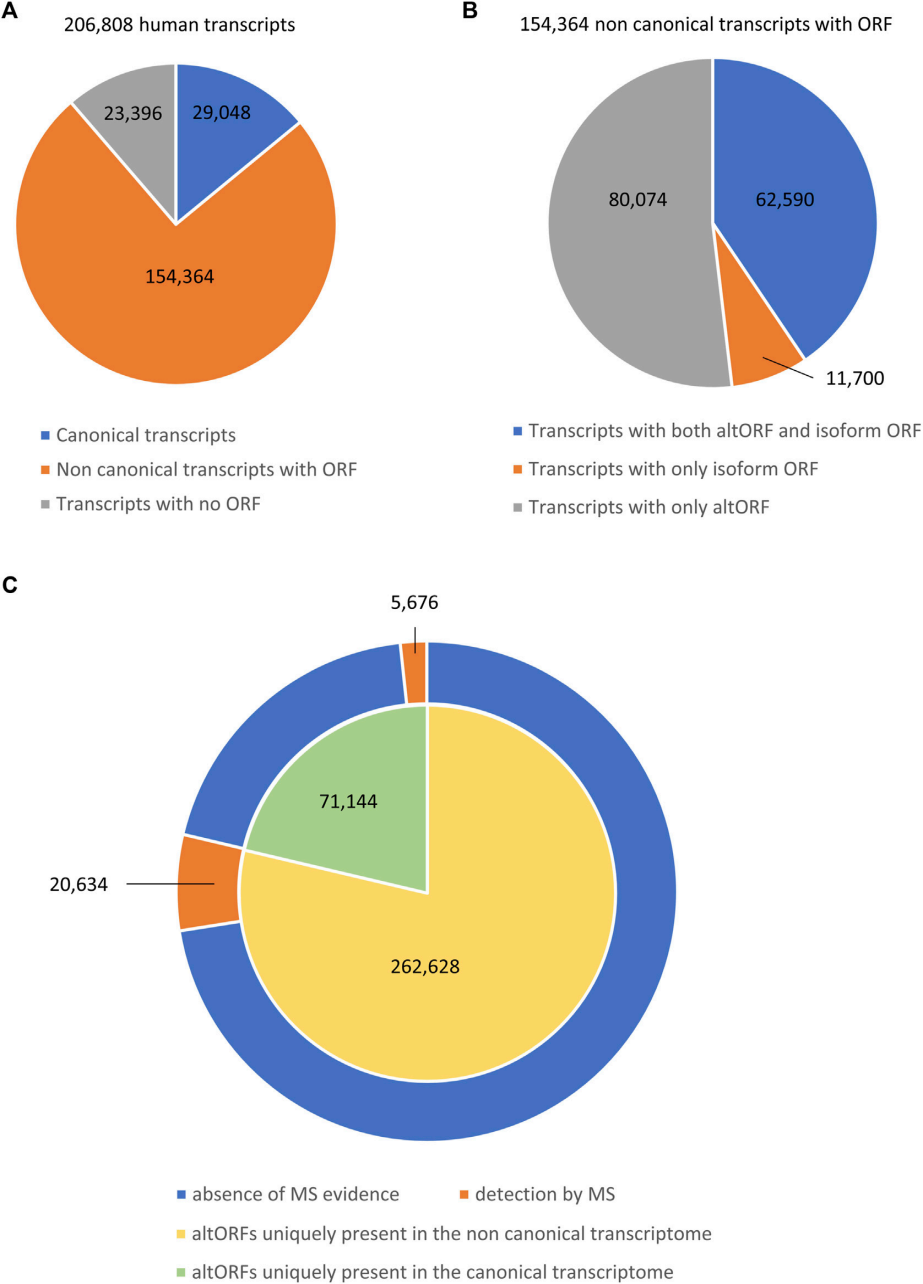
Composition of the human transcriptome. **(A)** Pie chart showing the number of different transcripts from the human reference genome (GRCh38 v95). Three types of transcripts are represented: canonical transcripts encoding a reference protein (blue), non-canonical transcripts generated through alternative splicing that contain an ORF (orange), and transcripts that do not have an annotated ORF (gray). **(B)** Pie chart showing the proportion of different sub-types of non-canonical transcripts containing an ORF. Three sub-types of transcripts are represented: non-canonical transcripts with both an alternative ORF (altORF) and an isoform ORF (blue), non-canonical transcripts with only an isoform ORF (orange), and non-canonical transcripts with only an altORF (gray). **(C)** Double pie chart representing the distribution of altORFs uniquely present in the canonical transcriptome (green) or the non-canonical transcriptome (yellow). Using the OpenProt database (Brunet et al., 2019), the evidence obtained by mass spectrometry (MS) of altORF-related proteins is represented in orange in the ring, while the absence of evidence is represented in blue.

A major problem is that alternative proteins expressed from these altORFs are usually not represented in the conventional protein databases (Brunet, Leblanc, and Roucou 2020; Cardon, Fournier, and Salzet 2021). Therefore, these alternative proteins represent a “ghost proteome” that was not considered until recently. Data-driven tools such as the sORF repository (Olexiouk, Van Criekinge, and Menschaert 2018) or the OpenProt database (Brunet et al., 2021a) have now been developed to offer a broader view of proteomes. The existence of thousands of altORFs hidden within known coding sequence of mRNAs raises the question of whether AS could also contribute to proteomic diversity through these small alternative proteins. To address this question, we performed a computational analysis using Ensembl human genome annotation (GRCh38 v95) and the OpenProt database (version 1.6) to determine the impact of AS on this hidden proteome. We identified a total of 206,808 transcripts including 29,048 transcripts defined as canonical as they encode reference proteins (Figure 1A). These transcripts might contain altORFs coding for alternative proteins. We also identified 154,364 transcripts (74.6%) that we categorized as non-canonical since they derive from AS but are not referenced to encode for reference proteins (Figure 1A). However, these transcripts may encode isoforms of reference proteins and/or contain an altORF. Finally, we identified 23,396 transcripts (11.3%) with no ORF according to the OpenProt database (Figure 1A). We next analyzed the non-canonical coding transcriptome landscape. Among these 154,364 transcripts, we identified 62,590 transcripts (40.5%) that contain both an ORF coding for an isoform of a reference protein and an altORF (Figure 1B). We found 80,074 transcripts (51.9%) only containing altORFs and 11,700 transcripts (7.6%) only containing an ORF coding for an isoform of a reference protein (Figure 1B). Our analysis highlights that AS generates numerous transcripts that do not encode for an isoform of a reference protein, supporting the claim by Tress and colleagues that AS might not be the key to proteomic complexity (Tress, Abascal, and Valencia 2017a). However, these transcripts contain altORFs that can potentially code for alternative proteins. These altORFs might also be commonly present in the related canonical transcripts as they could be located in the exons that are not directly affected by AS. We analyzed the distribution of these altORFs and identified 71,144 altORFs that were uniquely present in the canonical transcriptome (29,048 transcripts), while 262,628 altORFs were uniquely present in the non-canonical transcriptome (154, 364 transcripts) (Figure 1C). It represents an average of 2.4 unique altORFs per canonical transcript and 1.7 unique altORFs per non-canonical coding transcript. Using the OpenProt database that encompasses 87 ribosome profiling and 114 mass spectrometry studies from several species, tissues, and cell lines (Brunet et al., 2019), we looked for mass spectrometry evidence for all these altORFs. We found that 5,676 unique altORFs (7.98%) in canonical transcripts had evidence in mass spectrometry, while 20,634 unique altORFs (7.85%) in non-canonical transcripts produced alternative proteins detected by mass spectrometry (Figure 1C). This result clearly indicates that AS can indeed contribute to the human proteomic diversity through the translation of altORFs within mature RNAs.

### Contribution of long non-coding RNAs and circular RNAs

Long non-coding RNAs (lncRNAs) represent an important part of the transcriptome (Liu et al., 2005; Derrien et al., 2012). LncRNAs are transcripts of 200 nucleotides or more that should not harbor protein-encoding ORFs (Dinger et al., 2008; Khalil et al., 2009; Derrien et al., 2012). Genome-wide translation profiling has recently revealed that small ORFs identified in lncRNA genes can code for micropeptides, polypeptides with a length of less than 100 amino acids essential for cellular growth (Chen et al., 2020). Other small peptides produced from lncRNAs have also been reported in functional studies (Odermatt et al., 1997; MacLennan and Kranias 2003; Slavoff et al., 2013; Ruiz-Orera et al., 2014; Pang, Mao, and Liu 2018; Wang J et al., 2019; Hartford Corrine and Lal, 2020; Nita et al., 2021; Mise et al., 2022). Eukaryotic lncRNA genes are usually composed of multiple exons with an average of 2.49 exons per human lncRNA gene (Khan, Wellinger, and Laurent 2021). LncRNA transcripts are efficiently spliced with a very similar distribution of AS-type events to that of protein-coding transcripts (Khan, Wellinger, and Laurent 2021). Hence, lncRNAs also generate multiple splice variants whose functional relevance can be associated with RNA-based differential functions (Khan, Wellinger, and Laurent 2021). Although the majority of alternatively spliced lncRNAs are likely non-functional, some of them can produce micropeptides. Indeed, specific splice variants of lncRNAs have the unique capability to produce functional micropeptides that are not encoded by the lncRNA of reference, that is, HOXB-AS3 lncRNA (Huang et al., 2017), LINC00948 lncRNA (Anderson et al., 2015), and LINC00665 lncRNA (Guo et al., 2020). Therefore, the proteomic diversity also depends on AS of lncRNAs. With a total of 354,855 lncRNA genes identified in 17 different species, the exact contribution of lncRNA splice variants to the proteomic complexity remains to be precisely determined and will be a major challenge in the field.

Circular RNAs (circRNAs) are produced from the back-splicing of linear RNAs where upstream splice-acceptor sites are covalently linked to downstream splice-donor sites to form an RNA loop structure (Kristensen et al., 2019). CircRNAs can be conserved during evolution and exhibit a tissue- or cell-specific expression (Kristensen et al., 2019; Santer, Bär, and Thum 2019). CircRNAs are functionally important as they act as microRNA decoys or scaffolds that sequester specific proteins (Chen et al., 2020). Due to their circular shape, circRNAs were not predicted to be translated, but there is growing evidence that circRNAs containing small ORFs can produce micropeptides that have a functional relevance (Legnini et al., 2017; Pamudurti et al., 2017; Liang et al., 2019; Lei et al., 2020; Sinha et al., 2022). It has been hypothesized that AS, particularly exon skipping, drives the formation of circRNAs. However, *in silico* analyses of AS and circRNA production in the human heart revealed that only 10% of circRNAs are produced from alternatively spliced exons, while 90% of circRNAs come from constitutive exons (Aufiero et al., 2018). Therefore, it is possible that AS can also impact the proteomic composition *via* circRNAs containing small ORFs, even though this contribution probably remains limited since circRNAs are described to largely be non-functional products of splicing errors (Xu and Zhang 2021). Future studies on circRNA translation will help uncover the circRNA-driven hidden proteome and enlighten on the functional importance of these novel proteins.

## Perspectives

Although MS combined with long-read sequencing and ribosome profiling data has significantly improved the identification of new isoform proteins, many MS fragment spectra still remain unidentified and could potentially result from alternative proteins, micropeptides translated from lncRNAs, circRNAs, or other RNAs (Makarewich and Olson 2017). Moreover, identifying isoform proteins or small proteins using “bottom–up” MS is challenging. An alternative form of a protein must have a tryptic peptide with more than eight amino acids in the region that differs from the canonical protein to be identified correctly. In addition, this peptide must be suitable for ionization and fragmentation. For small proteins with less than 100 amino acids, the chance to have unique detected peptides is strongly reduced compared to large proteins. Size selection, enrichment of small-size proteins, and careful selection of proteases may improve detection of low abundant proteins and micropeptides. Furthermore, matching MS spectra with custom databases will also help successfully identify novel isoform proteins or small-size micropeptides. “Top–down” proteomics, which characterizes intact proteins in complex mixtures without prior digestion, could be a good alternative approach. However, this method requires long ion accumulation, activation, and detection times and has not been achieved on a large scale due to lack of methods integrated with tandem MS. Despite significant advances, identifying new isoform proteins in the proteome complexity remains a challenge, and further improvements (e.g., methodology, filtering criteria, and database) will be required to substantially improve this situation in the future.

Determining which alternatively spliced transcripts produce proteins with important biological functions (i.e., isoform proteins, alternative proteins, and micropeptides) is the key to confirm the real impact of AS on proteomic complexity. To date, relatively few isoform and alternative proteins have been studied at the functional level, and the biological significance of AS-derived proteome remains obscure. For some AS events, functional consequences can be easily inferred based on changes in the protein sequence. Some alternatively spliced transcripts can encode protein isoforms, which lose or gain specific domains. Interestingly, 50% of AS events in the human transcriptome preserve the ORF and 65% of these frame-preserving splice variants are detected in polysome fractions and, hence, are likely translated (Weatheritt, Sterne-Weiler, and Blencowe 2016). This observation indicates that alternatively spliced transcripts with no frame preservation are potentially eliminated by quality control processes such as NMD. Indeed, some AS events can lead to the inclusion of highly conserved “poison” exons, which contain a premature truncation codon (Leclair et al., 2020). Although these exons do not contribute to the protein-coding capacity, their AS coupled to NMD plays an autoregulatory role in gene expression and protein abundance. Hence, the functional consequences of AS are not always obvious, and many studies failed to detect any differences in the activity of isoform proteins. However, the absence of functional relevance does not mean that there are no functional differences. Therefore, determining the biological function of a single AS event or an AS-derived product will be a major challenge of the proteomic era in the upcoming years.

AS also has a strong clinical relevance since dysregulations of AS have been associated with many chronic diseases including cancer (Ouyang et al., 2021; Zhang et al., 2021). It is, therefore, critical to advance the functional characterization of the AS-derived proteome, but the identification of AS events without regard to their contribution to proteomic diversity is also essential. Indeed, it is key to further study any potential AS alterations in diseases or pathological conditions as they could be valuable prognostic and diagnostic biomarkers. Such investigations could also provide tools for the development of therapeutics. Two splicing-based therapeutic agents are currently tested in clinical trials: small-molecule splicing modulators and antisense oligonucleotides (ASOs). Small-molecule drugs modulate the splicing activity by directly targeting the spliceosome and splicing factors. Surprisingly, these compounds do not induce global splicing inhibition but rather selective changes in AS for genes related to cell proliferation and apoptosis (Folco, Coil, and Reed 2011; Vigevani et al., 2017). However, potential problems of off-target effects require that AS mechanisms are fully understood before further clinical use. In contrast, ASOs are emerging as more secure therapeutic agents to modulate splicing. ASOs can specifically neutralize splice sites, inhibit the recruitment of specific RNA-binding proteins or inhibit the expression of specific splice variants (Rinaldi and Wood 2018). For instance, clinical applicability of ASO-based strategies has been successful in the treatment of patients with spinal muscular atrophy (Hua et al., 2008). ASOs could be used to specifically target specific disease-related splice variants, but advancing knowledge on the functional roles of isoform proteins is, hence, critical for efficient clinical interventions. Regardless of its contribution to proteomic diversity, targeting AS is now recognized an important area for clinical intervention.

## Conclusion

On the contentious question “Does alternative splicing really expand proteomic diversity?,” we can hereby affirm that AS indeed participates to proteomic complexity in many ways, that is, isoform proteins, alternative proteins, and micropeptides. In the light of this re-evaluation, the AS-related ghost proteome fills a gap and enlarges our vision of the current proteome. Importantly, the remaining limitations on the original question should be taken in consideration in future research endeavors. To continue assessing AS contribution to proteomic complexity, deeper ORF annotation and improvement of technologies and methodologies will be key to functional proteomic discoveries. With a repertoire of alternatively spliced transcripts now significantly expanded, more extensive functional studies on AS and its related proteome are necessary to unravel their unexpected implications in a variety of biological processes.
